# The Homeostasis of Cartilage Matrix Remodeling and the Regulation of Volume-Sensitive Ion Channel

**DOI:** 10.14336/AD.2021.1122

**Published:** 2022-06-01

**Authors:** Zhiqin Deng, Xiaoqiang Chen, Zicong Lin, Murad Alahdal, Daping Wang, Jianquan Liu, Wencui Li

**Affiliations:** Hand and Foot Surgery Department, Shenzhen Second People's Hospital/the First Hospital Affiliated to Shenzhen University, Shenzhen 518000, China.

**Keywords:** osteoarthritis, chondrocytes, ion channel, cell volume regulation

## Abstract

Degenerative joint diseases of the hips and knees are common and are accompanied by severe pain and movement disorders. At the microscopic level, the main characteristics of osteoarthritis are the continuous destruction and degeneration of cartilage, increased cartilage extracellular matrix catabolism, decreased anabolism, increased synovial fluid, and decreased osmotic pressure. Cell volume stability is mainly regulated by ion channels, many of which are expressed in chondrocytes. These ion channels are closely related to pain regulation, volume regulation, the inflammatory response, cell proliferation, apoptosis, and cell differentiation. In this review, we focus on the important role of volume control-related ion channels in cartilage matrix remodeling and summarize current views. In addition, the potential mechanism of the volume-sensitive anion channel LRRC8A in the early occurrence of osteoarthritis is discussed.

## Introduction and epidemiology

Osteoarthritis (OA) is a disease that seriously impacts the health and quality of life of people, and its prevalence is increasing worldwide [1]. OA is the second most important risk factor for adult disability. Globally, the number of patients with advanced OA who need joint replacement is increasing due to the aging population.

**Figure 1.**
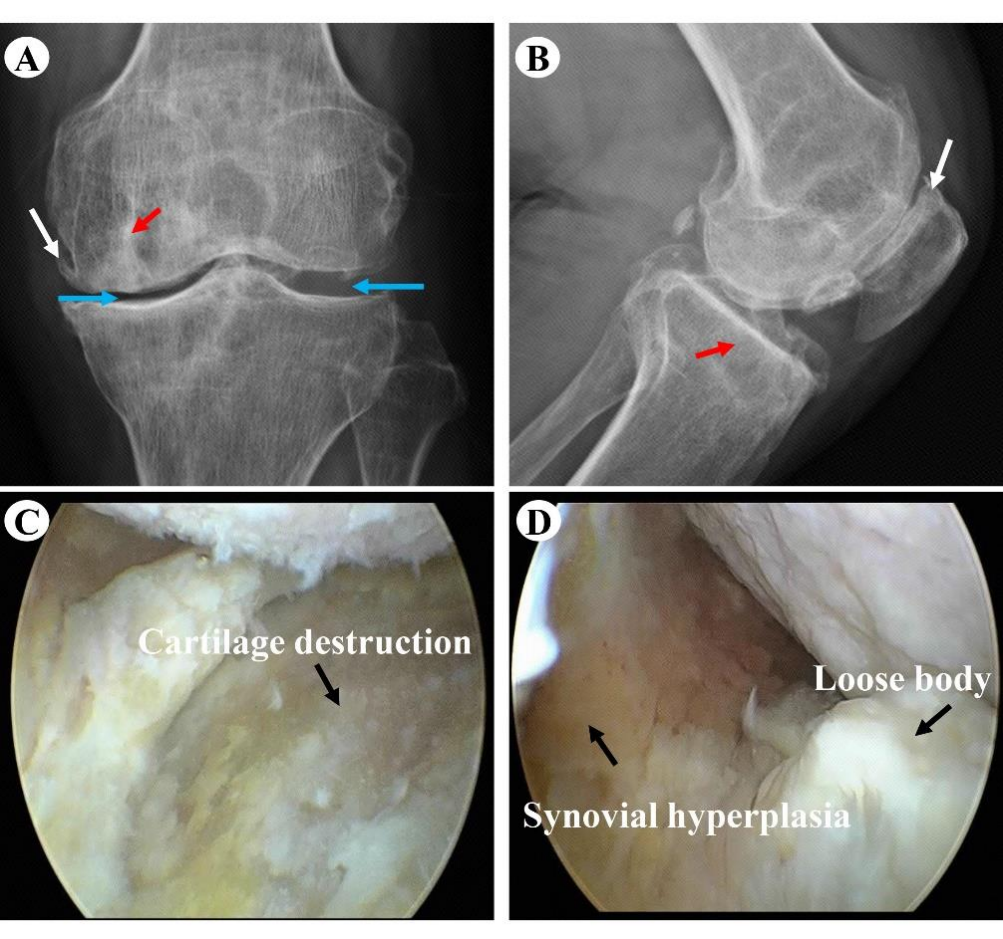
Radiographs and arthroscopy of osteoarthritis. *A* and *B* are anteroposterior and lateral radiographs, respectively. *C* and *D* are arthroscopy observations. A 57-year-old male patient came to see the doctor because a "sprain during exercise caused pain and discomfort in the left knee joint for one month." X-rays of the left knee showed narrowing of the medial space of the knee joint, hyperplasia of osteophytes, sclerosis of subchondral bone, and loose bodies in the intercondylar fossa. A CT scan was performed to further clarify the abovementioned lesions. Later, the patient underwent arthroscopic knee surgery. Intraoperative detection revealed that the cartilaginous surface of the left medial condyle of the femur and the medial plateau of the left tibia were severely damaged with arthritic synovial hyperplasia and a loose body in the intercondylar fossa. Red arrow: sclerosis of the subchondral bone in the left knee joint. Blue arrow: the internal joint space of the left knee is narrowed. White arrow: hyperplasia of osteophytes in the left knee joint.

The function of joints is determined by anatomy and biomechanics. Hip dysplasia is a congenital anatomical abnormality that can lead to acquired OA [2]. In addition, excessive physical activity is an important risk factor for arthritis caused by abnormal biomechanics (Fig. 1). However, physical activity is also beneficial. Appropriate running frequency and intensity, and adequate pre-exercise preparation are effective for cartilage protection [3, 4]. Various risk factors can lead to an imbalance of cartilage matrix synthesis and catabolism in articular cavity, which affects the occurrence and development of OA. Thus, reversing the imbalance of cartilage matrix metabolism is important in the prevention and treatment of OA. Cell volume homeostasis allows cells to perform their basic physiological functions. Significant decreases in osmotic pressure of synovial fluid in patients with OA can trigger cellular inflammatory signals and disrupt the metabolic balance of the cartilage matrix. However, the mechanisms underlying decreases in the osmotic pressure of synovial fluid are still unclear. Hence, there is a need to (i) improve our understanding of the imbalance of cartilage matrix synthesis, and (ii) catabolism, and develop safe and effective new drugs for the prevention and treatment of OA.

### Reversing the imbalance of cartilage matrix remodeling is important in the prevention and treatment of OA

The loss of cartilage matrix integrity is a typical feature of OA. However, the molecular mechanisms underlying the imbalance of cartilage matrix synthesis/catabolism that lead to OA are still unclear. Reversing the metabolic synthesis/catabolism imbalance of cartilage matrix remodeling is key to maintaining cartilage integrity. Articular cartilage is mainly composed of chondrocytes and extracellular matrix. The cartilage matrix is composed of the proteoglycan aggrecan (Acan), collagen type II (COL II), and matrix metalloproteinases (MMPs). Chondrocytes and the extracellular matrix together maintain the normal structure and function of articular cartilage [5, 6]. Obesity, mechanical force, and other factors promote the occurrence of inflammation in the joint cavity. Many inflammatory factors such as interleukin-1β (IL-1β) and tumor necrosis factor alpha (TNF-α) upregulate the expression of MMP-13, which in turn triggers an increase in matrix catabolism, disrupts the balance between cartilage matrix synthesis and catabolism, and leads to cartilage degenerative diseases, such as OA. We recently confirmed that increased expression of COL I, decreased expression of COL II, a disordered cytoskeletal protein distribution, and loss of cell membrane integrity are important characteristics of OA, indicative of a decrease of anabolism of human OA chondrocytes [7]. We also confirmed that high expression of MAPK-14 may promote chondrocyte death [8]. Another study found that among several important proteoglycan proteases involved in matrix catabolism, the expression levels of MMP and aggrecanases (ADAMTs) are significantly higher in OA synovial fluid and joint tissues. Several selective inhibitors for ADAMTS-4 and ADAMTS-5 have been clinically tested for OA, but their efficacy has not been confirmed. Therefore, the development of a strategy for reversing the imbalance between cartilage matrix synthesis and catabolism to prevent and treat OA is still crucial.

### Decreased osmotic pressure of synovial fluid is an important risk factor for OA

A decrease in the osmotic pressure of synovial fluid can lead to an increase in cellular inflammatory signals, which may be an important risk factor for OA. Studies have confirmed that the osmotic pressure of the synovial fluid of OA patients is reduced to (297.3 ± 16.9 mOsm/L). Although this is the osmotic pressure of normal cell culture medium, it is much lower than the osmotic pressure in the joints of people without OA (404 ± 57 mOsm/L) [9]. Research has shown that extracellular hypotonicity is an important danger signal. Cell volume homeostasis is closely related to physiological activities such as metabolism, cell proliferation, and apoptosis. However, when the change in osmotic pressure exceeds the threshold of cellular self-regulation, cell volume homeostasis is disrupted, triggering a series of pathological changes. The low osmotic pressure of the extracellular fluid activates NLRP3 inflammasomes and caspase-1 and induces the release of the inflammatory factor IL-1β [10]. The nucleotide-binding oligomerization domain-like receptor family pyrin domain-containing 3 (NLRP3) inflammasome is the main sensors of cellular sensor osmotic pressure and causes the release of IL-1β [11].

Clinically, the osmotic pressure of saline used in arthroscopic surgery ranges from 250-300 mOsm/L, which may also be an important factor that further aggravates chondrocyte damage [9]. A recent clinical study showed that intra-articular injection of platelet-rich plasma is superior to injection with hyaluronic acid (osmotic pressure 300 mOsm/L) or normal saline in the treatment of moderate knee OA [12]. This result suggests that normal saline may be a potential risk factor for OA because it is “hypotonic” compared with the osmotic pressure in the normal joint cavity (400 mOsm/L). Hypertonic saline can reduce scalpel-induced cartilage cell death in cattle and rats by about 50% [13]. An important feature of OA is tissue swelling and enhanced hydration, which leads to a decrease in the osmotic pressure of the synovial fluid and a change in the composition of ions. The levels of Ca^2+^, Fe^2+^, K^+^, Mg^2+^, and other cations in synovial fluid all increase [14]. In addition, the drop in osmotic pressure of synovial fluid further aggravates the electrolyte imbalance in the joint cavity, leading to swelling and death of tissue cells. However, the underlying cause of the drop in osmotic pressure of synovial fluid is still unclear.

### Sustained hypotonicity causes mitochondrial damage and cartilage matrix metabolism imbalance

The decrease of osmotic pressure in the joint cavities of OA patients is a continuous and gradual process. When chondrocytes are exposed to risk factors such as hypotonicity for a long time, the cells swell and possibly die. Hypotonic cells can undergo rapid change in the structural states of their organelles. Mitochondria have a dynamic structure. In a static state, the average inter-membrane distance between the inner and outer mitochondrial membranes is relatively narrow short. Under stress, the mitochondrial matrix shrinks, the distance between the membranes increases, and the mitochondrial volume increases. The volume homeostasis of the mitochondrial matrix is regulated by potassium channels [15, 16]. Adenosine triphosphate (ATP)-sensitive potassium channels are composed of ATP pore-forming (MITOK) and ATP-binding (MITOSUR) subunits. Overexpression of MITOK triggers swelling of organelles, and low expression can cause instability of the mitochondrial membrane potential[17]. In addition, the voltage-dependent anion channel (VDAC) and chloride intracellular channel (CLIC) proteins CLIC1, CLIC4, and CLIC5 have been identified to be mainly located in cardiac mitochondria[18-21]. However, water channels and chloride channels that have important regulatory effects on chondrocyte mitochondrial volume homeostasis have rarely been investigated. The response of chondrocyte mitochondria to hypotonic changes is closely related to the participation of ion channels. There is currently no evidence that ion channels with increased expression directly participate in the regulation of cartilage matrix metabolism by regulating mitochondrial function. However, there is additional evidence that the voltage-dependent anion channels VDAC1 and VDAC2 are expressed in chondrocytes [22], and the expression increases after exposure to IL-1β) and TNF-α) [23]. VDAC recruits ubiquitin ligase Parkin to defective mitochondria to promote mitochondrial autophagy, which has been shown to reduce OA [24, 25]. The volume homeostasis of mitochondria, which relies on ion transport across the inner membrane, is a key issue in cell pathophysiology. Some evidence points out that the large conductance K^+^ (BK) channel activated by Ca^2+^ opens when calcium ions in the mitochondrial matrix increase [26]. The large conductance K^+^ (BK) channel activated by Ca^2+^ has been confirmed to increase its expression in OA chondrocytes [22]. However, whether the BK channel-mediated regulation of mitochondrial volume homeostasis directly participates in cartilage matrix metabolism requires more evidence.

Research indicates that mitochondria fuse and divide in response to cellular needs and the environment. Changes in mitochondrial dynamics are the basis of many human diseases, such as cancer, neurological diseases, and cardiovascular diseases [27]. Metabolic stress that damages mitochondria can trigger mitochondrial fragmentation, leading to mitochondrial dysfunction [28]. Mitochondrial fragmentation is also related to mitochondrial dysfunction, such as mitochondrial DNA (mtDNA) mutations [29]. However, mitochondrial fusion is believed to promote processes such as oxidative phosphorylation [30]. Mitochondrial dysfunction is usually related to mitochondrial fission/fragmentation mediated by dynamin-related protein 1 (Drp1) [31]. AMPK can directly and rapidly promote mitochondrial fragmentation. Screening of AMPK substrates has identified the mitochondrial fission factor (MFF), the outer membrane receptor of mitochondrial DRP1 [28]. Drp1 and MFF are key proteins that mediate mitochondrial fission. The unbalanced processing of the mitochondrial fusion protein optic atrophy 1 (OPA1) or mitofusin 1 (MFN1) accelerates mitochondrial fission [32].

**Figure 2.**
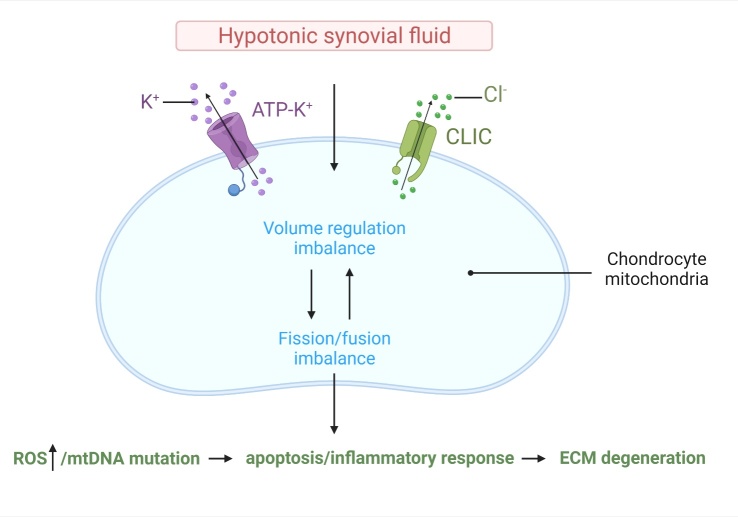
Continuous hypotonicity causes mitochondrial damage and cartilage matrix metabolism imbalance. When chondrocytes are in a hypotonic state for a long time, the imbalance of ATP-K and CLIC plasma channel function mediates the imbalance of mitochondrial volume regulation and fission and fusion in chondrocytes, leading to increased reactive oxygen species levels, mtDNA damage, chondrocyte apoptosis and inflammation. Eventually this leads to the destruction of the extracellular cartilage matrix.

Mitochondria generate reactive oxygen species, which are closely related to age-related mitochondrial dysfunction. Mitochondrial dysfunction leads to an imbalance between the production of reactive oxygen species and the antioxidant capacity of cells, which is an important factor in the development of OA [33-35]. Evidence shows that mitochondrial quality, the mitochondrial DNA content of chondrocytes, and the levels of electron transport chain proteins and proteins involved in mitochondrial biogenesis are reduced in patients with OA [36] (Fig. 2).

### Hypotonicity causes inflammation

**Figure 3.**
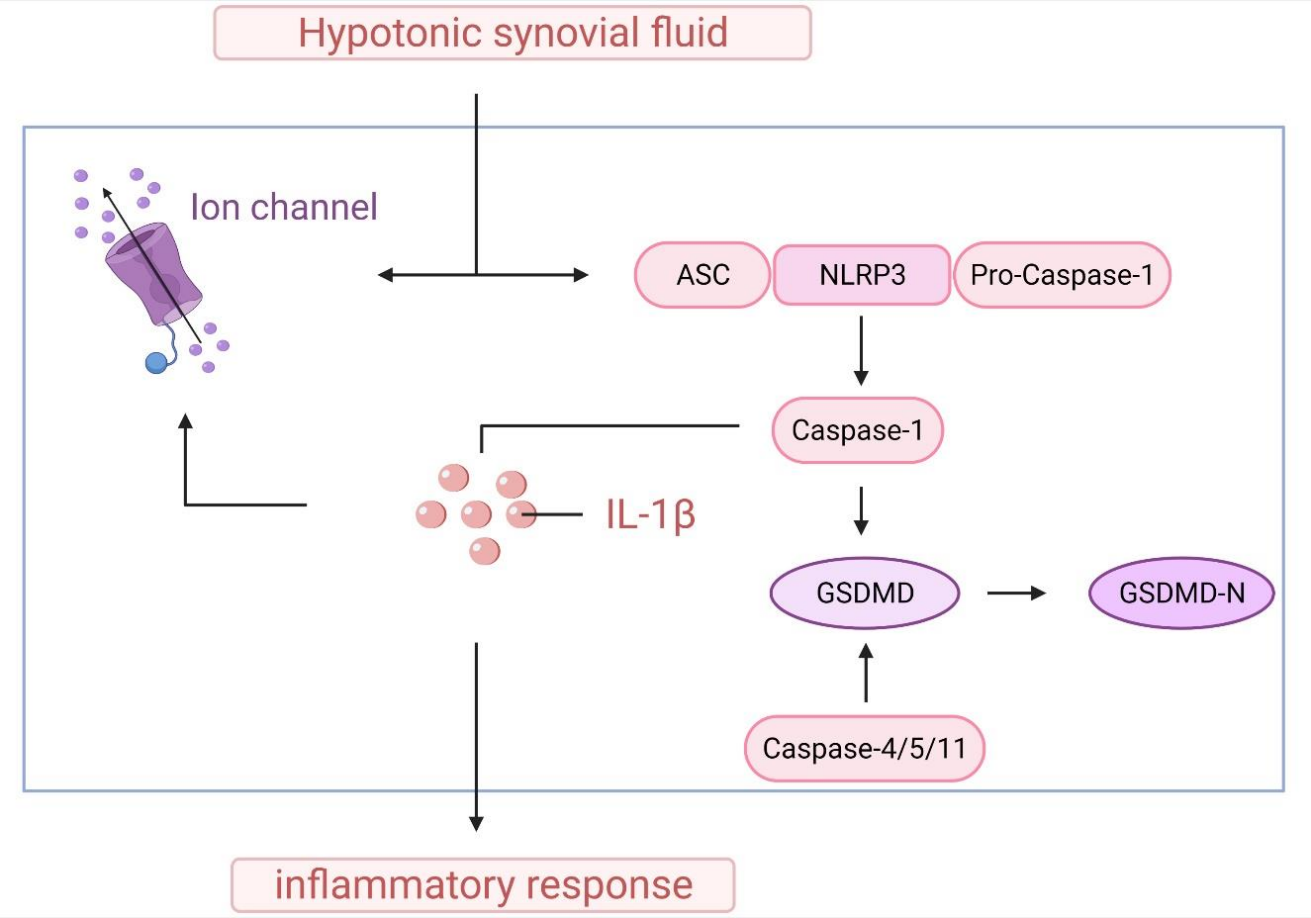
The process of inflammation caused by hypotonic cells. Extracellular hypotonicity activates ion channels on chondrocytes, increases the outflow of K^+^ and Cl^-^, and modulates NLRP3 inflammasome and apoptosis-related dot-like protein ASC oligomerization, which induces the maturation of pro-caspase-1. Finally, mature caspase-1 induces the release of large amounts of IL-1β. At the same time, caspase-1 and caspase-4/5/11 specifically cleave GSDMD and trigger cell inflammation.

Pyrolysis, also known as cell inflammatory necrosis, is a kind of programmed cell death that manifests as an increase in cell volume and continuous expansion until the cell membrane ruptures, which leads to the release of cell contents and activates a strong inflammatory response [37]. Caspases are a family of cysteine proteases that play an important role in apoptosis and the regulation of inflammation. Caspase-1 and caspase-11 are jointly involved in the process of pyrolysis [38, 39]. A study have confirmed that caspase-1 and caspase-4/5/11 specifically cleave the linker between the amino-terminal gasdermin-N and C-terminal gasdermin-C domains in gasdermin D (GSDMD), which is necessary and sufficient for the regulation of inflammatory necrosis [40]. Activated caspase-1 and/or caspase-11 aggregate in large quantities and activate IL-1β and IL-18. A large number of activated inflammatory factors eventually lead to inflammatory cell death, that is, pyrolysis [38]. The swelling or shrinking of cells is accompanied by the activation of ion channels. In the process of pyrolysis, the cell volume continues to increase, the membrane is lysed and dissolved, and the cell undergoes programmed death, which may be closely related to the activation of ion channels [41]. Studies have reported that hypotonicity induces cell swelling, which in turn activates the release of caspase-1 and IL-1β through the NLRP3 inflammasome [10]. Uric acid crystals can activate NLRP3 inflammasomes and trigger IL-1β secretion, suggesting that inflammasome activation and IL-1β secretion are the driving factors of gout [42]. IL-1β deficiency or IL-1β blockers can improve atherosclerosis in mice [43]. Cholesterol crystals can activate the NLRP3 inflammasome, and its activation promotes atherosclerosis in low-density lipoprotein receptor-deficient mice. In addition, islet amyloid polypeptide (IAPP) can activate the secretion of IL-1β in insulin cells [44]. The activation of NLRP3 inflammasomes is an important step in the inflammatory death of chondrocytes. In addition, it has been reported that Cl^-^ efflux can dynamically induce the oligomerization of apoptosis-related dot-like protein ASC and activate the NLRP3 inflammasome [45], further confirming the key role of the opening or closing of anion channels in regulating cell inflammation (Fig. 3).

**Table 1:** Ion channels and related functions in chondrocytes.

	channels	Ion transduction	Steps impacted	Role in chondrocytes	Ref.
1	Na, K ATPase-sodium pump.	Na^+^, K^+^	Na^+^, K^+^-ATPase correlates with matrix glycosaminoglycan concentrations	extracellular matrix formation; mechanotransduction (?)	[59, 60]
2	Na V 1.8	Na^+^	activation of NaV1.8 mediates OA pain related DRG neuron hypersensitivity	pain regulation	[61]
3	ENaC: epithelial sodium channel;	Na^+^	inhibits RVI by reducing the influx of Na+ ions through ENaC/Deg-like ion channels act as a possible target for chondrocyte volume.	cell volume regulation	[62]
4	BK Ca Calcium-Dependent Potassium Channels	K^+^	Activation of ion channels by membrane stretch	mechanotransduction and chemotransduction.	[63]
5	TRPV4(non-selective cation channel)	Ca^2+^	TRPV4-mediated Ca^2+^ signaling in the response of chondrocytes to physiologic levels of strain	mechanotransduction	[56, 64, 65]
6	PIEZO channels	Ca^2+^	directly activated by mechanical stress	mechanotransduction	[64, 66]
7	T-type VGCCs	Ca^2+^	inhibition of chondrocyte T-type VGCCs reduces Ca^2+^ responsiveness by ~50%	mechanotransduction	[67, 68]
8	VDCCs:voltage-dependent calcium channel;	Ca^2+^	activation of VDCC function appears to accompany various inflammatory aspects of osteoarthritis	Inflammation regulation	[69, 70]
9	P1/P2: purinergic receptors;	Ca^2+^	Piezo1 and Piezo2 act as transduction channels for high-strain mechanical stress in chondrocytes	mechanotransduction	[66]
10	SOCE: store-operated Ca^2+^ channel entry	Ca^2+^	Inhibition of SOCE combined with ER calcium store depletion abolished differentiation and severely diminished proliferation in chondrifying mesenchymal cells	chondrocyte differentiation and proliferation	[71]
11	NMDAR: N-methyl- D-aspartate receptors;	Ca^2+^	NMDAR inhibition resulted in reduced MMP13 and COL10A1 in osteoarthritic chondrocytes, but increased MMP13 and COL10A1 in macroscopically normal chondrocytes	chondrocyte clock,mechanotransduction;proliferation;early chondrogenesis	[72-75]
12	Acid-sensing ion channels (ASICs)	H^+^	activation of ASIC1a-dependent JAK2/STAT3 and MAPK/NF-κB signaling pathways enhance chondrocyte apoptosis	pain regulation	[76]
13	clc-3	Cl^-^	Swelling-activated ClC-3 regulates prostaglandin E2 release	cell volume regulation	[77]
14	ClC-7	Cl^-^	increase [Ca^2+^]i and cell death.	cell death regulation	[54]
15	VDAC: voltage-dependent anion channel	Cl^-^	VDAC1 acts as mitochondrial docking sites to recruit PRKN from the cytosol to the damaged mitochondria, involved in mitochondrial damage, which may be related to chondrocyte autophagy	Mitophagy	[24, 25]
16	CFTR	Cl^-^	Functional expression of cystic fibrosis transmembrane conductance regulator in mouse chondrocytes.	not clear	[78]
17	SLC26A2	Cl^-^	SLC26A2 mutations resulting in defective sulfate uptake in chondrocytes	mutation causes osteochondrodysplasia	[79, 80]
18	pannexin	-	Pannexin 3 regulates intracellular ATP/cAMP levels and promotes chondrocyte differentiation	chondrocyte differentiation	[81, 82]

### The relationship between increased expression of water channels and OA

Aquaporin-1 (AQP-1) is distributed in normal human tissues, including cardiovascular, respiratory tract, cartilage, and other connective tissues [46]. The cells in the nucleus pulposus and inner fibrous annulus of the human intervertebral disc express both AQP-1 and AQP-3, but not AQP-2 [47]. Water channels are an important part of cell membranes. Water channels, cation channels, and anion channels work together to control (i) ion transport inside and outside the cell and (ii) volume homeostasis. They also play a role in osmotic changes in synovial fluid in OA. Recent studies have pointed out that AQP-1 expression in OA chondrocytes is 39.8 times higher than in non-OA chondrocytes [22]. Down-regulation of AQP-1 reduces the expression of ADAMTS-4 in human chondrocytes, which may inhibit the catabolism of cartilage matrix [48]. AQP-1 water channels are highly expressed in OA chondrocytes and closely related to the expression and activity of caspase-3 [48, 49]. By knocking out AQP-1 in chondrocytes *in vivo* by RNA interference, it was found that the expression and activity of caspase-3 in chondrocytes cultured *in vitro* were significantly decreased, suggesting that high expression of AQP-1 promotes chondrocyte apoptosis [50].

### The expression and function of ion channels on chondrocytes

Ion channels and transporters in many types of cells (including chondrocytes) mediate reductions and increases in volume [51, 52]. Various types of ion channels have been identified on the plasma membranes of mammalian chondrocytes: voltage-dependent Na^+^ channels, epithelial Na^+^ channels, transient receptor potential channels, voltage-dependent K^+^ channels, and Cl^-^ channels [53, 54]. In articular chondrocytes, voltage-dependent K^+^ channels and double-hole domain K^+^ channels help maintain the resting membrane potential [55]. TRPV4-mediated Ca^2+^ signaling in the response of chondrocytes to physiologic levels of strain suggests that targeting Piezo2-mediated mechanotransduction, which is induced by injurious and repetitive mechanical stimulation, is feasible for OA treatment [56]. In Table 1, the ion channels related to various functions in chondrocytes are summarized. These ion channels are closely related to mechanical transduction, inflammation regulation, volume regulation, and cartilage differentiation, and they all play important functions in the regulation of chondrocytes. In OA, it is accompanied by a large number of changes in ion channels. A data analysis of 10 Affymetrix microarray data sets indicated that the expression of KCNMA1, KCNN4, and TMEM16A increased in OA chondrocytes, while the expression of KCNK5, KCNT2, and SCNN1A decreased significantly [22]. The expression of CLC-7 in response to changes in osmotic pressure decreased in human chondrocytes. Short-term culture (48 hours) in a hypotonic medium (270 mOsm/L) will reduce the expression of ClC-7 and reduce the acid-sensitive current, and lead to increased chondrocyte death [54]. In addition, TRPV4 channel helps in the early stage of hypotonic stress, while BK(Ca) channel participates in the increase of intracellular Ca^2+^ and mediates cell volume reduction [57]. Mice lacking the TRPV4 channel have severe arthritis changes [58].

The surface of articular cartilage contains only a small number of chondrocytes (1-10% of the total tissue volume in mature cartilage) in addition to a large amount of cartilage matrix components. However, chondrocytes are the only cells in articular cartilage. Chondrocytes are constantly affected by various stresses, including mechanical load stimulation, changes in the osmotic pressure of synovial fluid, and various endogenous substances (including inflammatory factors and exosomes) in physiological and pathological states. Effective functional homeostasis of chondrocytes is very important. Failure of various ion channels in chondrocytes to function properly may cause the occurrence and development of OA.

### The relationship between chondrocyte volume adjustment and cartilage matrix remodeling

**Figure 4.**
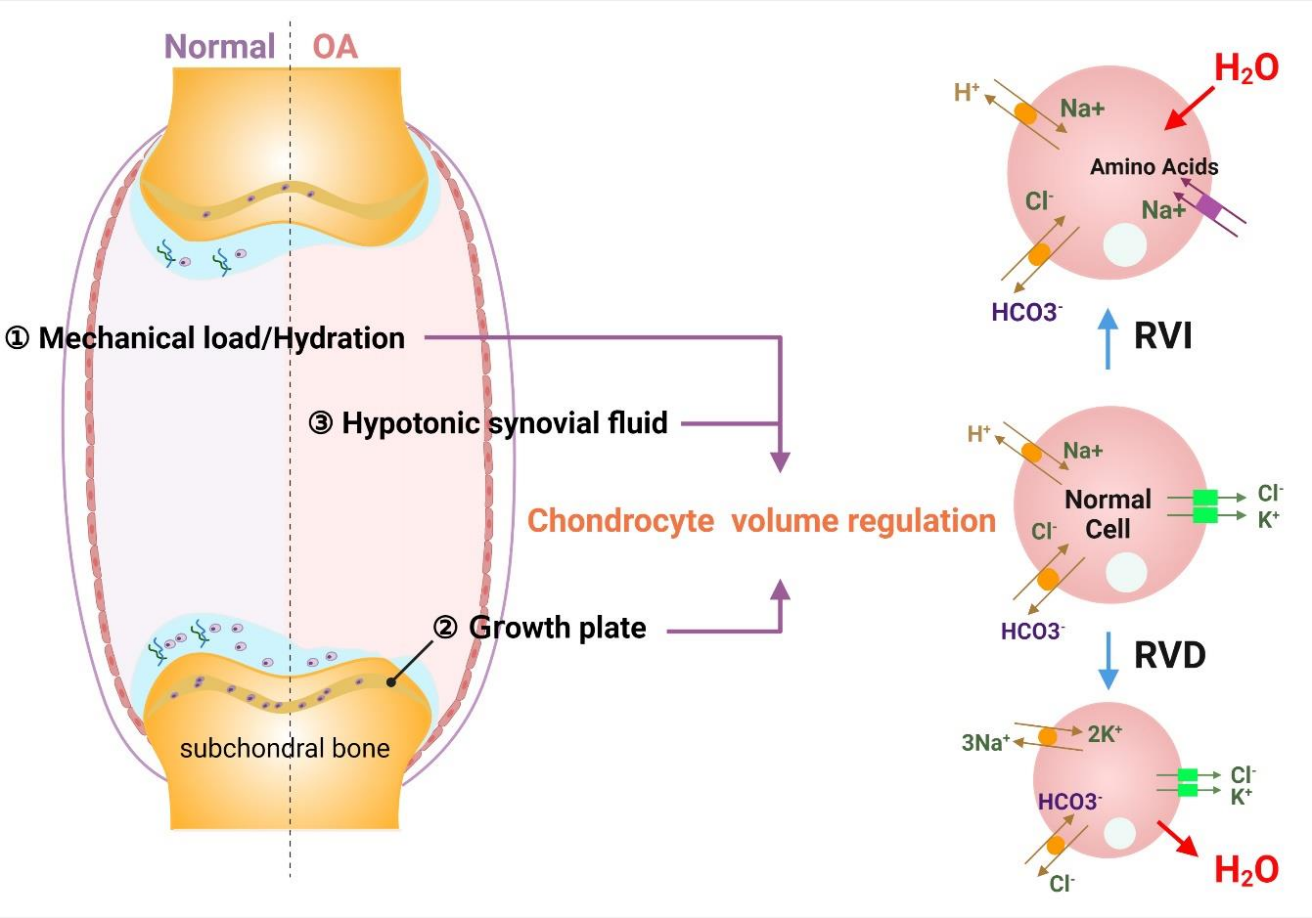
Volume regulation of chondrocytes in physiological or pathological environments. First, under physiological conditions, the cartilage matrix is hydrated by mechanical loads such as exercise, and the osmotic pressure of synovial fluid is in a physiological state that regulates chondrocyte volume and maintains chondrocyte viability. Second, under physiological conditions, growth plate hypertrophic chondrocytes become physiologically enlarged, and endochondral ossification occurs. Third, in a pathological state, the synovial fluid is persistently hypotonic, and the capacity of chondrocyte volume regulation is weakened, leading to other pathological processes. The regulation of chondrocyte volume is jointly dominated by RVI and RVD, accompanied by the activation of a large number of ion channels and the transport of ions across the membrane.

Volume regulation of chondrocytes is essential to cartilage matrix metabolism homeostasis. Cartilage cell volume regulation can occur in different physiological or pathological environments [83]. First, with mechanical loads such as exercise, hyaline cartilage changes hydration, resulting in continuous changes in the synovial fluid osmotic pressure in a physiological environment, to which chondrocytes are constantly exposed. Second, growth plate hypertrophic chondrocytes become physiologically enlarged in the process of endochondral ossification [84, 85]. Third, a large number of studies have shown that in the pathological state of arthritis, increased water content causes the synovial fluid to be persistently hypotonic, which increases the risk of chondrocyte death and matrix loss (Fig. 4). During volume homeostasis, the regulation of volume decrease (RVD) and regulation of volume increase (RVI) processes interact to maintain a stable chondrocyte volume under physiological conditions, supporting cartilage function and extracellular matrix stability. Under physiological conditions, cells cannot always expand or shrink, and cell volume is regulated by the volume homeostasis mechanism. RVD is a process of active volume-regulating retraction after cells swell rapidly in a hypotonic solution. This process is regulated by a series of ion channels and transporters, such as Na^+^, K^+^, and Cl^-^ channels. RVD is also found in chondrocytes. For example, Lewis et al. observed that the TRPV5-regulated positive resting membrane potential may be a protective adaptive mechanism in chondrocyte volume regulation [86]. Inactivation of the volume-sensitive outward rectification (VSOR) current under acidic conditions can impair the volume regulation of chondrocytes, which may reduce the viability of chondrocytes [53]. RVI mostly occurs in a hypertonic environment. Chondrocytes can also undergo RVI [87, 88]. Studies have also shown that intracellular calcium [Ca^2+^]i does not participate in RVD or RVI. The activation of RVI is instead regulated by Na^+^-K^+^-2Cl^-^ cotransporters[88, 89] (Fig. 4).

Volume homeostasis of chondrocytes is a primary condition for their function. Studies have shown that changes in extracellular osmotic pressure lead to changes in volume that have an important impact on the integrity of the cartilage matrix [90, 91]. Our team found that in human OA chondrocytes, the cytoskeletal protein distribution is disorderly, and the levels of cartilage matrix proteins such as COL II are significantly decreased [7]. Other studies have found that in an early OA model of rabbits, the cartilage matrix is not yet lost, but the chloride channels have changed, accompanied by a decrease in the ability of chondrocytes to regulate their volume [92]. Cl^-^ conductance in chondrocytes is thought to be involved in the synthesis of extracellular matrix in addition to the regulation of the resting membrane potential [54]. As an important candidate volume-sensitive chloride channel, leucine-rich repeat-containing protein 8A (LRRC8A) is involved in many pathophysiological processes. One study using a YFP quenching assay and whole genome small RNA molecule (21-25 nucleotides) scanning of mammalian cells identified LRRC8A as the main component of the volume-regulated anion channel (VRAC) [93, 94]. However, the role of the volume-sensitive LRRC8A chloride channel in the regulation of cartilage matrix metabolism requires more research.

### Ion channels mediate the disruption of internal and external circulation in osteoarthritis

Here, we view OA progression as a joint process of internal circulation and external circulation, and we discuss the roles that ion channels play. The volume of *in situ* chondrocytes in the surface and middle layers of articular cartilage has been found to increase with cartilage degeneration [83, 95]. However, it is still unclear whether chondrocyte volume control imbalance, mitochondrial damage, apoptosis, and inflammation are factors that induce cartilage degeneration or byproducts of cartilage destruction. What is certain is that once a process is abnormally regulated, this will inevitably lead to other reactions and cartilage matrix degeneration, forming a vicious circle, leading to advanced OA. For example, as discussed above, when the extracellular osmotic pressure drops, this can trigger an inflammatory response. The activation of NLRP3 inflammasomes and the formation of ASC dot-like protein are regulated by Cl^-^ [10, 45]. The inflammatory factor IL-1β, which activates the chondrocyte chloride current, may be involved in cartilage matrix metabolism [7], which is a progressively aggravated pathological process that is regulated by a variety of factors. Among them, the mutual regulation of water channels, ion channels, and inflammatory factors is crucial.

**Figure 5.**
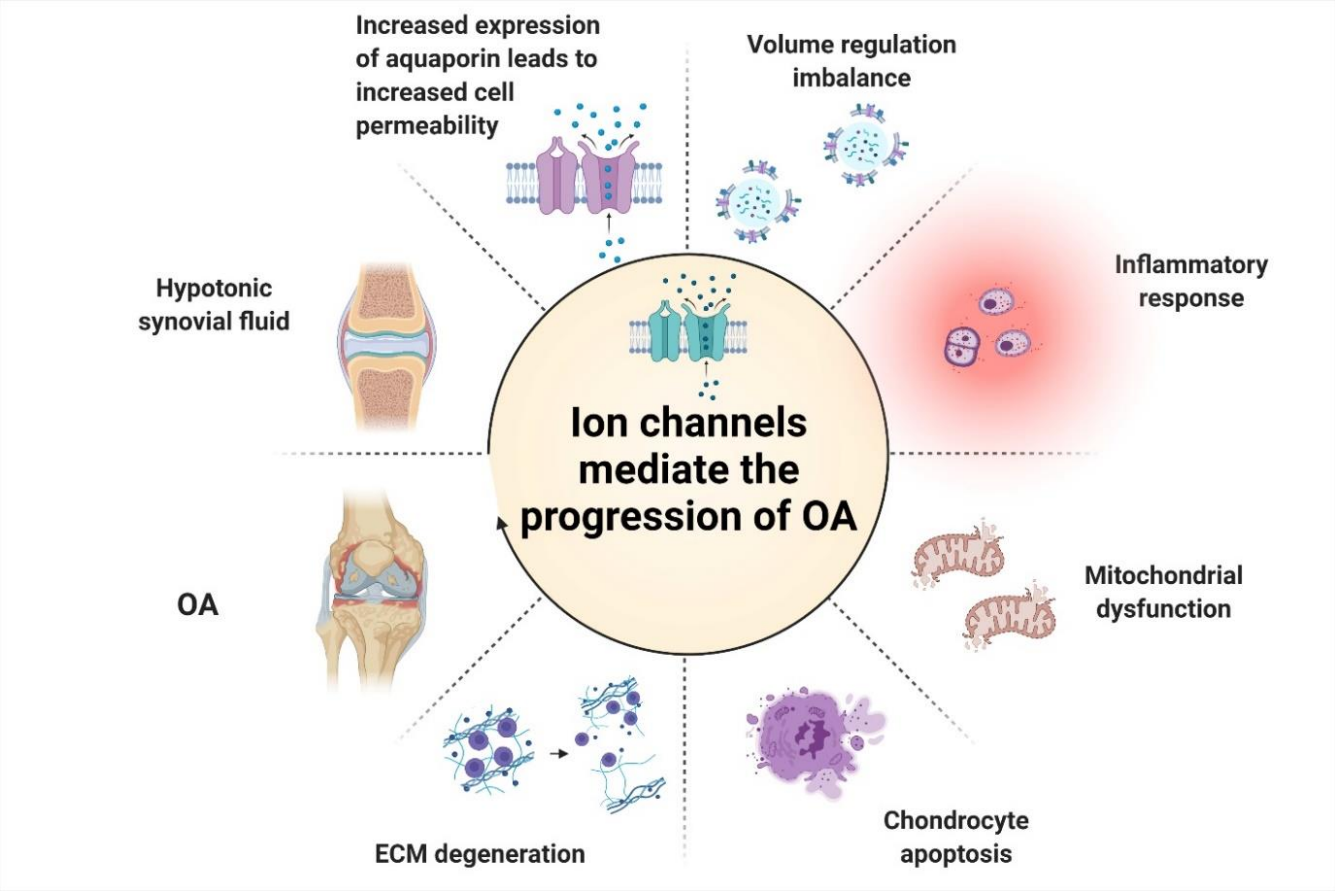
The internal and external circulation of osteoarthritis. Internal circulation: in chondrocytes, increased expression of aquaporin, chondrocyte volume control imbalance, mitochondrial damage, apoptosis, and inflammation are interrelated and interact to destroy chondrocytes. External circulation: the destruction of chondrocytes, the increase of cartilage matrix degradation, and the decrease of osmotic pressure of synovial fluid result in osteoarthritis.

When chondrocytes are pathologically damaged, the cartilage matrix degenerates and the osmotic pressure of synovial fluid further decreases, resulting in destruction from the inside out. In addition, when the osmotic pressure of the synovial fluid continues to decrease, or the cartilage matrix degenerates due to excessive load, this will aggravate chondrocytes, which is a pathological change of OA from the outside to the inside. For example, Lee and colleagues used viscoelastic hydrogels in cartilage tissue and found that faster relaxation was associated with a significant increase in the volume of the interconnected cartilage matrix formed by chondrocytes. In slower relaxation gels, the restriction of elastic stress on cell volume expansion leads to increased secretion of IL-1β, which in turn promotes the strong upregulation of genes related to cartilage degradation and cell death [96] (Fig. 5).

### Conclusion and future perspectives

OA increasingly threatens affects the health of the elderly and even the young. However, it is a complex disease with many factors influencing its development. In the treatment of OA it is important to effectively reverse the imbalance of cartilage matrix synthesis/catabolism. To maintain chondrocyte volume regulation homeostasis, mitochondrial function stability, chondrocyte apoptosis, and orderly inflammatory responses are goals in the treatment of OA. Studies have confirmed that increased expression of AQP-1 water channels in OA chondrocytes causes increased chondrocyte permeability [22]. In addition, the continuous decrease of local osmotic in the OA articular cavity causes mitochondrial dysfunction, inflammation, chondrocyte death and cartilage matrix metabolism imbalance. These pathological changes of chondrocytes are regulated and involved in volume-sensitive ion channels. At present, it is not clear that the specific molecular mechanism of cartilage degeneration caused by ion channels involved in chondrocyte volume control imbalance, mitochondrial damage, cell apoptosis, and inflammation. However, to reverse the imbalance of cartilage matrix metabolism, it is necessary to establish a new and effective method to block the continuous decrease of the osmotic pressure of the joint cavity and cause the imbalance of chondrocyte volume control. LRRC8A may be an important target among them.

LRRC8A is a key volume-sensitive chloride channel that is closely related to tumor prognosis [97], the inflammatory response [98, 99], superoxide production[100], and many other physiological and pathological functions. The volume-sensitive anion channel is activated in response to hypotonic pressure. Closely related paralogues of the LRRC8 family are the main components of volume-regulating anions [93, 94], which assemble together to form hexameric complexes [101]. In OA synovial fluid, what is the effect of the continuous hypotonic synovial fluid environment on the volume-sensitive anion channel on chondrocytes, especially whether there is a functional change in the LRRC8A chloride channel? Some studies have pointed out that in an early OA model of rabbits, the cartilage matrix has not yet begun to be destroyed, but it has been found that the chloride channel has changed, and the ability of chondrocyte volume regulation is reduced [92], does it mean that LRRC8A is also inactivated? Does the inactivation of LRRC8A chloride channel mediate the decrease of chondrocyte volume regulation ability? The increase in AQP-1 expression, the dysfunction of Cl^-^ channels, and the decrease in volume regulation may represent the early stage of OA. In response to these series of issues, put forward these points: First, change the local osmotic pressure environment of the articular cavity in the early stage of OA to delay cartilage matrix degeneration. Second, whether it is possible to enhance the osmotic pressure microenvironment regulation of the articular cavity by enhancing the function of LRRC8A. Further clarification of these issues may provide an important treatment method for OA, even early OA.
